# Clinical, laboratory and pathological findings in sub-acute monensin intoxication in goats

**Published:** 2014

**Authors:** Mahdi Deljou, Mohammad Reza Aslani, Mehrdad Mohri, Ahmad Reza Movassaghi, Mohammad Heidarpour

**Affiliations:** 1 *Department of Clinical Sciences, School of Veterinary Medicine, Ferdowsi University of Mashhad, Mashhad, Iran;*; 2 *Department of Clinical Sciences, Faculty of Veterinary Medicine, Shahrekord University, Shahrekord, Iran; *; 3 *Department of Pathobiology, School of Veterinary Medicine, Ferdowsi University of Mashhad, Mashhad, Iran.*

**Keywords:** Cardiomyopathy, Goat, Monensin, Oxidative stress, Poisoning

## Abstract

Toxic effects of monensin, a polyether antibiotic mainly used as a coccidiostat, have been described in a wide range of animals. The present study was performed to investigate the toxic effects of monensin in goats. Seven adult goats were administered sodium monensin, 13.5 mg kg^-1^, daily for five consecutive days via gastric gavage. Monensin toxicity was evaluated by clinical signs, serum biochemistry and pathology. Monensin exposure caused diarrhea, tachycardia and reduction in ruminal movements and body temperature. Significant increase of creatine kinase, lactate dehydrogenase, aspartate aminotransferase, total bilirubin, blood urea nitrogen, creatinine and erythrocyte superoxide dismutase were observed in monensin exposed goats. Reduction of erythrocyte glutathione peroxidase and elevation of serum malondialdehyde and troponin I were inconsistent. In necropsy, there were effusions in body cavities, vacuolar degeneration and coagulative necrosis in cardiac and skeletal muscles and renal tubular necrosis. These findings suggested that monensin intoxication in goats leads to cardiac, skeletal and renal damage and a wide range of biochemical abnormalities. Oxidative stress may be involved in the pathogenesis of monensin poisoning.

## Introduction

Monensin is an antibiotic produced as a byproduct of fermentation by *Streptomyces cinnamonensis* and belongs to a family of drugs known as polyether antibiotics or ionospheres. Monensin was the first antibiotic that showed an effect at practical concentrations for incorporation in feed as an anticoccidial agent.^1^ However, in recent years it has been widely used as a feed additive to improve performance in livestock production systems. Monensin has also been used in the treatment and prevention of ketosis, lactic acidosis, bloat and acute pulmonary edema and emphysema.^2^

Monensin has a low therapeutic index and may be fatal in certain species when used in excessive doses. Accidental monensin intoxication, usually occur after mixing errors that result from its inclusion in feeds of non-target species or in excessive concentration in the diets of target species.^3^ Monensin intoxication has been reported in cattle^4^^, ^^5^ water buffaloes,^6^ sheep,^7^^,^^8^ horses,^9^ swine,^10^ chickens,^11^ ostriches,^12^ deer,^13^ and dogs.^14^^,^^15^ Monensin has been approved for use in non-lactating goats.^16^ However, there are no published reports of field cases of monensin toxicosis in goats. Experimental data indicated that the single oral LD_50_ dose for monensin in goats is 26.4 mg kg^-1^ of body weight.^17^

Susceptibility to monensin toxicity varies considerably between species. Horses are unusually sensitive to monensin and other ionophores intoxication and fish are the most tolerant to high levels of ionophores. The LD_50_ of monensin in horses is as low as 1.4 mg kg^-1^, while its LD_50_ for chicken, the least sensitive species, is 214.0 mg kg^-1^.^3^ Monensin toxicity may also be potentiated by concurrent use of various antibiotics, including tiamulin, oleandomycin chloramphenicol and macrolides, and sulfa drugs.^3^^,^^4^

In this study, clinical, laboratory and pathological features of sub-acute monensin intoxication have been investigated in goats.

## Materials and Methods

**Animals and treatments. **Seven clinically healthy local-breed, female, non-lactating and non-pregnant goats weighing 35-40 kg and aged 2-4 years were purchased from a local market. Prior to commencement of the experiment, goats received 0.2 mg kg^-1 ^of subcutaneous ivermectin (Razak Co., Tehran, Iran,) and 7.5 mg kg^-1 ^of oral rafoxanide (Daru, Tehran, Iran) and were kept in indoor pen and fed for 14 days to ensure proper acclimation. Fresh water was available all the time. Monensin (Razak Co., Tehran, Iran) was administered orally via orogastric tube at the dose of 13.0 mg kg^-1^ body weight daily for five days.

**Blood collection and serum biochemistry. **Venous blood samples with and without anticoagulant were collected for hematology and measurement of total plasma protein and serum concentrations of troponin I, albumin, blood urea nitrogen (BUN), creatinine, total antioxidant capacity (TAC), activities of creatine kinase (CK), aspartate amio transferase (AST) and lactate dehydrogenase (LDH) on day 0 and afterward daily until day 10. The total protein was determined by refractometry and the fibrinogen concentration was measured by the heat-precipitation refractometry method. Other biochemical parameters were measured by an automated biochemical analyzer (Model Targa 3000, Biotecnica, Rome, Italy) using commercial kits for assessment of iron, CK, AST, LDH, BUN, creatinine, bilirubin and albumin (Pars Azmoon, Tehran, Iran) and for TAC (Randox, Antrim, UK). Serum troponin I concentration was determined using an ELISA kit (Monobind Inc, Lake Forest, USA). 

The concentration of malondialdehyde (MDA) was estimated in serum according to the method of Placer *et al*.^18^ The reaction mixture consisted of 0.20 mL of serum, 1.30 mL of 0.20 M Tris, 0.16 M KCl of buffer (pH 7.4) and 1.50 mL of thiobarbituric acid reagent. The mixture was heated in a boiling water bath for 10 min. After cooling, 3 mL of pyridine/n-butanol (3:1, v/v) and 1.00 mL of 1M sodium hydroxide were added and mixed by vigorous shaking. A blank was run simultaneously by incorporating 0.20 mL distilled water instead of the serum. The absorbance of the test sample was read at 548 nm. The nmol of MDA per ml of serum was calculated using 1.56 × 10^5^ as extinction coefficient. 

The activities of erythrocyte glutathione peroxidase (GPx) and superoxide dismutase (SOD) were determined in erythrocyte hemolysate obtained immediately after sampling from the blood with anticoagulant. Activity of GPx was measured using commercially available kits (Ransel test kit; Randox Laboratories Ltd., London, UK). This method is based on that of Pagalia and Valentine.^19^

Activity of SOD was measured based on a modified method of iodophenyl nitrophenol phenyl tetrazolium chloride (INT; Randox Laboratories Ltd., London, UK). One unit of SOD causes a 50% inhibition of the rate of reduction of INT under the assay conditions. 

**Pathological examinations. **The goats were immediately necropsied after sacrificing on day 10 and all macroscopic changes of different organs were recorded. To examine microscopic lesions, tissue samples of heart, liver, kidneys and skeletal muscles (quadriceps muscles) were collected, fixed in 10% neutral buffered formalin and processed for routine histology. Tissue sections were stained with hematoxylin and eosin (H & E) for light microscopic examination.

**Statistical analysis. **Statistical analysis was conducted using SPSS for windows (Version 16; SPSS Inc., Chicago, USA). Based on Kolmogorov–Smirnov normality test, non - parametric Friedman and Wilcoxon Signed tests were used to compare significant differences within group for measured parameters. For all comparisons, *p* < 0.05 was considered as significant. For controlling the rate of type I error, the P value of each pairwise comparison was multiplied by the number of comparisons.

## Results

**Clinical findings of monensin toxicosis in goats. **Clinical signs of toxicity such as diarrhea and tachycardia were observed in monensin exposed goats. Monensin caused significant elevation of heart rate and a significant decrease in ruminal contraction. Body temperature was significantly decreased during the first four days of the experiment and then returned to baseline values. No significant change was observed in the respiratory rate.

**Effect of monensin on serum troponin I, CK, AST and LDH. **Significant increase in serum troponin I concentration was observed on days 3, 4, 7 and 8 of the study with a median peak concentration of 2.03 ng mL^-1^ on day 8. The CK and LDH activities were significantly elevated in all timepoints compared to baseline values. Significant increase in activity of serum AST was observed on days 8, 9 and 10 (Table 1).

**Table 1:** Changes of serum aspartate aminotransferase, creatine kinase, lactate dehydrogenase activities in the goats intoxicated with monensin.

**Day**	**Aspartate aminotransferase (IU L** ^-1^ **)**				**Creatine kinase (IU L** ^-1^ **)**				**Lactate dehydrogenase (IU L** ^-1^ **)**			
	**25** ^th^ *	**50** ^th^ *	**75** ^th^ *	***p*** **	**25** ^th^	**50** ^th^	**75** ^th^	***p*** **	**25** ^th^	**50** ^th^	**75** ^th^	***p*** **
**0**	69.00	77.00	92.00	-	160.00	278.00	301.00	-	324.00	505.0	559.00	-
**1**	64.00	75.00	85.00	0.270	187.00	274.00	1166.00	0.043	670.00	737.00	838.00	0.018
**2**	54.00	68.00	77.00	0.236	198.00	313.00	549.00	0.028	613.00	624.00	764.00	0.018
**3**	61.00	78.00	93.00	0.463	249.00	570.00	791.00	0.028	572.00	611.00	820.00	0.018
**4**	68.00	84.00	100.00	0.735	245.00	410.00	605.00	0.018	514.00	649.00	759.00	0.028
**5**	72.80	87.50	100.50	0.249	210.80	332.50	387.80	0.046	496.30	560.50	722.00	0.075
**6**	48.50	88.00	95.80	0.752	276.00	489.00	576.80	0.028	502.80	602.50	838.30	0.207
**7**	69.50	142.00	774.00	0.075	257.00	446.00	22233.00	0.028	574.50	646.00	3314.80	0.144
**8**	91.00	156.00	2388.50	0.043	355.30	654.00	14973.00	0.028	665.00	810.50	3043.50	0.058
**9**	128.50	194.00	4292.80	0.028	296.80	674.00	6010.00	0.028	543.30	809.00	1183.30	0.115
**10**	129.80	214.50	5837.30	0.028	275.80	950.50	13080.00	0.028	943.30	943.50	2270.30	0.400
***p*** ***	0.008				0.000				0.000			

* 25^th^, 50^th^ and 75^th^ percentiles;

** *p-*value for each day compared to baseline value;

*** Overall *p*-value.

**Effect of monensin on serum MDA, TAC and erythrocyte SOD and GPx. **Significant elevation of erythrocyte SOD activity was observed on all days compared to baseline value. Serum MDA levels were significantly increased on days 1 and 8 of the experiment. Significant decrease of erythrocyte GPx activity was observed on days 2 and 5. No significant change was observed in serum TAC level (Tables 2 and 3). 

**Table 2:** Effect of monensin on erythrocyte superoxide dismutase and glutathione peroxidase in the goats

**Day**	**Glutathione peroxidase (IU g** ^-1^ ** Hemoglobin)**				**Superoxide dismutase (IU g** ^-1^ ** Hemoglobin)**			
	**25** ^th^	**50** ^th^	**75** ^th^	***p*** **	**25** ^th^	**50** ^th^	**75** ^th^	***p*** **
**0**	32.50	43.95	48.07	-	3210.11	3333.33	3352.94	-
**1**	27.55	38.97	44.65	0.034	4118.55	5324.07	7717.39	0.018
**2**	26.64	29.36	37.50	0.735	5331.46	8137.25	14285.71	0.018
**3**	37.00	43.36	59.50	1.000	4687.50	5941.17	9513.88	0.018
**4**	30.05	36.28	48.43	0.176	6352.94	8000.00	8168.27	0.018
**5**	23.55	27.76	36.83	0.072	7907.63	9463.60	10418.50	0.028
**6**	30.20	33.44	44.80	0.027	6093.75	7596.68	9096.35	0.028
**7**	31.77	36.74	42.10	0.028	5977.49	7239.50	8623.45	0.028
**8**	32.23	39.63	49.40	0.058	5526.85	6443.62	6619.42	0.028
**9**	36.55	39.44	49.26	0.074	4663.74	5463.62	6964.62	0.028
**10**	33.72	39.25	42.30	0.917	4433.33	5658.33	6804.53	0.028
***p*** ***	0.013				0.000			

* 25^th^, 50^th^ and 75^th^ percentiles;

** *p-*value for each day compared to baseline value;

*** Overall *p*-value.

**Table 3:** Effect of monensin on serum malondialdehyde and total antioxidant capacity in the goats

**Day**	**Malondialdehyde ** **(nmol mL** ^-1^ **)**				**Total antioxidant capacity (mmol L** ^-1^ **)**			
	**25** ^th^ *	**50** ^th^ *	**75** ^th^ *	***p*** **	**25** ^th^	**50** ^th^	**75** ^th^	***p*** **
**0**	4.57	6.86	8.21	-	0.18	0.31	0.60	-
**1**	7.32	8.92	11.66	0.04	0.35	0.42	0.64	0.64
**2**	4.34	5.71	8.23	0.86	0.19	0.48	0.58	0.600
**3**	4.57	7.09	9.38	0.12	0.32	0.42	0.58	0.463
**4**	4.57	5.03	7.32	0.12	0.25	0.40	0.80	0.753
**5**	4.69	8.35	9.49	0.17	0.25	0.35	0.51	0.753
**6**	5.14	7.32	9.44	0.22	0.23	0.37	0.62	0.600
**7**	3.49	5.60	9.60	0.75	0.47	0.66	0.89	0.345
**8**	7.26	9.15	14.47	0.46	0.35	0.53	0.77	0.116
**9**	4.63	6.98	13.32	0.34	0.20	0.48	0.65	0.249
**10**	5.20	6.06	15.33	0.24	0.39	0.59	0.97	0.225
***p*** ***	0.395				0.425			

* 25^th^, 50^th^ and 75^th^ percentiles;

** *p-*value for each day compared to baseline value;

*** Overall *p*-value.

**Effect of monensin on serum albumin, bilirubin and plasma total protein. **Serum bilirubin concentrations were significantly elevated on all days after monensin administration when compared with baseline levels. Monensin administration had no significant effect on plasma total protein, fibrinogen and serum albumin concentration during the study period in comparison with baseline values (Table 4).

**Table 4:** Effect of monensin on plasma total protein and serum albumin and bilirubin in the goats

**Day**	**Total Protein (g dL** ^-1^ **)**				**Albumin (g dL** ^-1^ **)**				**Bilirubin (mg dL** ^-1^ **)**			
	**25t** ^th^ *	**50** ^th^ *	**75** ^th^ *	***p*** **	**25** ^th^	**50** ^th^	**75** ^th^	***p*** **	**25** ^th^	**50** ^th^	**75** ^th^	***p*** **
**0**	7.60	7.80	7.90	-	3.60	3.80	3.90	-	1.26	1.28	1.35	-
**1**	8.40	9.30	9.40	0.018	3.90	4.10	4.50	0.018	1.28	1.33	1.34	0.446
**2**	7.90	8.50	9.10	0.034	3.70	3.90	4.30	0.336	1.34	1.37	1.38	0.027
**3**	7.50	8.10	8.70	0.176	3.70	3.80	4.00	0.680	1.36	1.39	1.42	0.028
**4**	7.40	7.90	8.20	0.485	3.40	3.90	4.10	0.494	1.35	1.40	1.43	0.018
**5**	7.60	7.90	8.40	0.416	3.60	3.80	3.90	0.416	1.37	1.37	1.40	0.027
**6**	7.60	8.10	8.40	0.080	3.70	3.80	4.10	0.317	1.35	1.39	1.46	0.027
**7**	7.80	8.20	8.70	0.046	3.80	4.00	4.10	0.141	1.37	1.40	1.45	0.028
**8**	7.60	8.70	9.10	0.080	3.80	4.00	4.50	0.206	1.37	1.40	1.43	0.027
**9**	7.40	7.90	8.50	0.684	3.60	3.80	4.00	0.892	1.38	1.40	1.44	0.028
**10**	7.00	8.10	8.50	0.596	3.60	3.80	4.00	0.414	1.35	1.37	1.43	0.044
***p*** ***	0.002				0.005				0.005			

* 25^th^, 50^th^ and 75^th^ percentiles;

** *p-*value for each day compared to baseline value;

*** Overall *p*-value.

**Effect of monensin on serum levels of BUN, creatinine and serum troponin I. **Significant increase of serum BUN concentration was observed on days 1 to 4. The concentration of serum creatinine was significantly increased on days 1, 6 and 7. Monensin exposure ended up significant elevation of serum troponin I concentration on days 3, 8 and 9 in goats, (Table 5).

**Table 5:** Effect of monensin on serum blood urea nitrogen, creatinine and troponin I in the goats

**Day**	**Blood urea nitrogen (mg dL** ^-1^ **)**				**Creatinine (mg dL** ^-1^ **)**				**Troponin I (ng mL** ^-1^ **)**			
	**25t** ^th^ *	**50** ^th^ *	**75** ^th^ *	***p*** **	**25** ^th^	**50** ^th^	**75** ^th^	***p*** **	**25** ^th^	**50** ^th^	**75** ^th^	***p*** **
**0**	10.00	22.00	28.00	-	0.96	0.98	1.08	-	0.06	0.19	0.25	-
**1**	38.00	46.00	50.00	0.018	1.16	1.25	1.34	0.034	0.06	0.44	1.19	0.176
**2**	43.00	48.00	55.00	0.018	0.93	1.04	1.10	0.735	0.06	0.13	0.88	0.672
**3**	31.00	47.00	51.00	0.018	0.92	1.00	1.05	1.00	0.31	0.38	0.44	0.041
**4**	32.00	35.00	48.00	0.028	0.91	0.95	0.98	0.176	0.13	0.88	2.62	0.116
**5**	24.50	34.00	45.00	0.075	0.89	0.92	1.08	0.072	0.00	0.25	1.93	0.400
**6**	22.30	25.50	33.00	0.207	0.79	0.89	0.93	0.027	0.05	0.28	0.95	0.236
**7**	21.50	25.50	31.50	0.144	0.79	0.86	0.92	0.028	0.19	0.97	2.18	0.075
**8**	26.50	28.50	31.30	0.058	0.85	0.93	0.99	0.058	0.66	2.03	3.53	0.028
**9**	25.00	28.50	30.00	0.115	0.78	0.89	0.95	0.074	0.30	0.69	4.83	0.028
**10**	22.50	27.00	-	0.400	0.85	1.07	1.22	0.917	0.19	0.47	5.81	0.115
***p*** ***	0.000				0.000				0.094			

* 25^th^, 50^th^ and 75^th^ percentiles;

** *p-*value for each day compared to baseline value;

*** Overall *p*-value.

**Fig. 1:**
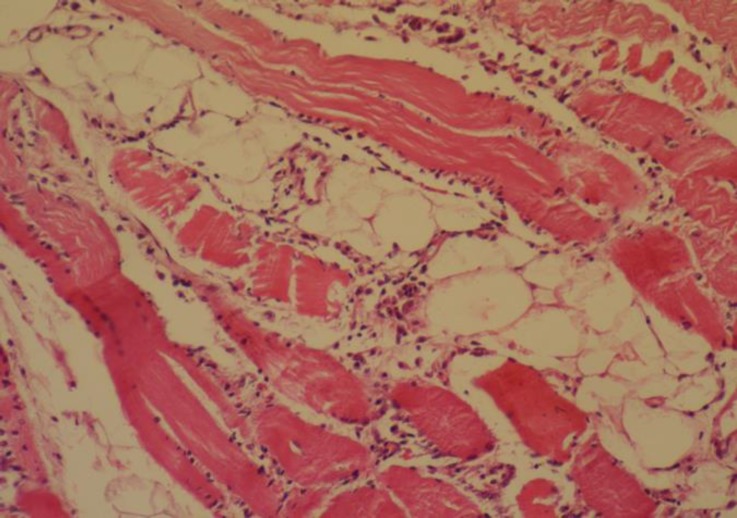
Severe coagulative necrosis in skeletal muscle (H & E, 320×).

**Fig. 2:**
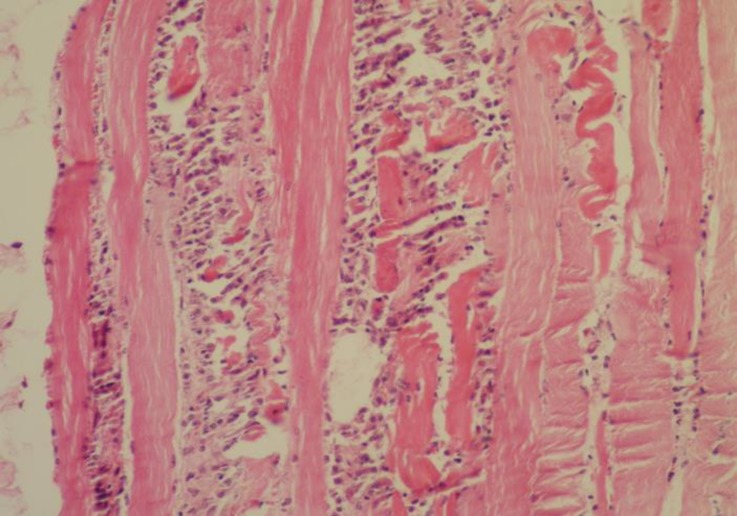
Severe coagulative necrosis in skeletal muscle associated with heavy infiltration of inflammatory cells (H & E, 320×).

**Fig. 3:**
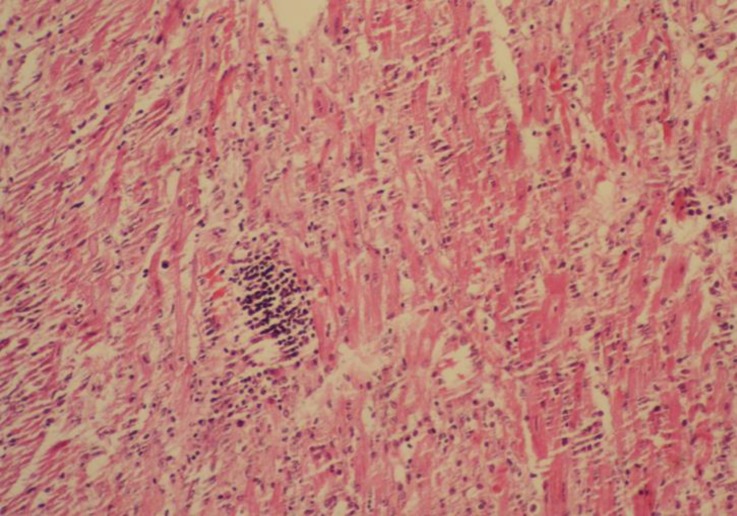
Severe multifocal necrosis in cardiac muscle cells with infiltration of inflammatory cells (H & E, 320×).

**Fig. 4:**
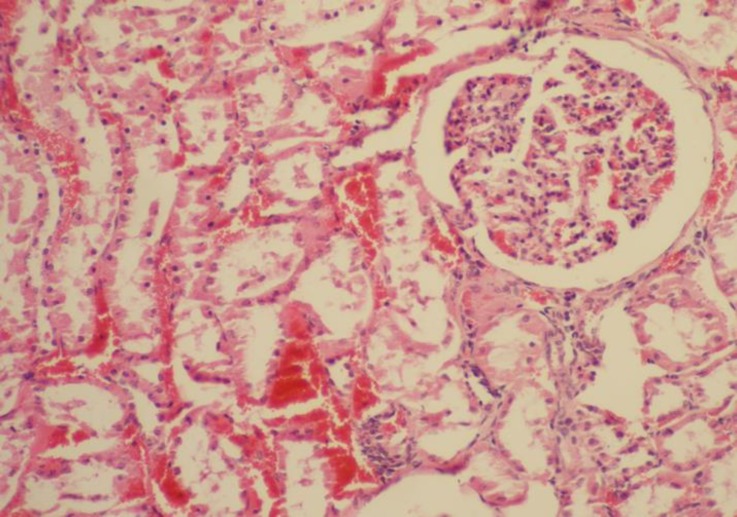
Severe congestion associated with renal tubular necrosis in affected kidney (H & E, 320×).

**Pathological findings. **At necropsy, the goats showed pulmonary edema, mild pleural, pericardial and peritoneal effusions, congestion of the liver and kidneys, pale area on the heart and skeletal muscles and sub-endocardial and sub-epicardial hemorrhages. Histopathology revealed severe coagulative necrosis in skeletal muscles which was associated with infiltration of mononuclear inflammatory cells, (Fig. 1). Vacuolar degeneration and severe multifocal necrosis were seen in cardiac muscle, (Figs. 2 and 3). The kidneys showed congestion and renal tubular necrosis, (Fig. 4). Congestion and hemosiderin–laden cells were observed in the liver.

## Discussion

Pathological and serum biochemical findings of sub-acute monensin intoxication in goats were relatively comparable to those of the previous descriptions of experimental and accidental monensin toxicosis in cattle, horses and sheep.^4^^,^^5^^,^^8^^,^^9^^,^^20^ These findings emphasize the potential role of monensin to cause widespread pathological changes in skeletal and cardiac muscles, as the primary target tissues. 

Monensin is a sodium selective ionophore which causes influx of sodium into the cells and produces higher intracellular concentrations of sodium. A rise in intracellular sodium in turn increases the intracellular levels of calcium due to an ATPase-driven exchange at the cell membrane and triggers the release of calcium from intracellular stores. Elevated calcium levels then activate muscle proteases and phospholipases which initiate degenerative processes in striated muscles and which ultimately lead to cell death.^3^ A direct role of lipid peroxidation and membrane perturbation during monensin toxicity has also been suggested.^21^

Observations to date indicate that in horses, the heart is the main target and shows greatest damage in monensin intoxication with little or no involvement of skeletal muscles. In contrast, in dogs and pigs lesions are more pronounced in skeletal muscle. Cattle, chickens and rodents have about equal predilection for cardiac and skeletal muscle lesions.^3^ According to the findings of the present study sub-acute monensin intoxication in goats seems to induce same lesions in both cardiac and skeletal muscles. Toxic nephrosis associated with monensin intoxication observed in goats of the present study, has also been described in the experimentally induced monensin poisoning in horses.^22^ and in cattle which has concurrently been received monensin and macrolide antibiotics^4^ but it has not been described in other reports.^6^^,^^8^^,^^23^^,^^24^^,^^25^ Hepatic lesions associated with accumulation of hemosiderin pigments observed in the current study, have rarely been reported in monensin intoxicated animals.^24^ Hemosiderosis was also observed in renal sections in the present study. 

Clinicopathological changes induced by monensin in goats were mainly consistent with cardiac and skeletal muscle damage. Serum troponin I is a highly sensitive and specific biomarker of myocardial damage in human medicine.^26^ It has also been studied in several laboratory and domestic animals.^27^ Detection of serum troponin I has not been reported in goats. Elevation of serum troponin I in cattle and horses with monensin toxicosis has been reported as a diagnostic tool for myocardial injury.^20^^,^^25^ In the present study, in spite of myocardial necrosis in goats received monensin, elevation of serum troponin I was not a consistent finding during the sampling period. On the other hand, noticeable and consistent increase in serum CK and LDH activities with lesser extent in serum AST activity, indicated progressive muscle structural damage in monensin exposed goats. These enzymes are normally localized in the cytoplasm and released into the circulation after the occurrence of cellular damage.^28^ Elevation of serum CK, AST and LDH has been reported in an accidental salinomycin, another ionophore compound, intoxication in Angora goats.^29^ Increases in serum concentration of bilirubin, BUN and creatinine which observed in the present study may be related to hepatic and renal dysfunction respectively and are considered as common biochemical abnormalities associated with monensin intoxication in other animals.^14^^,^^30^^,^^31^

The antioxidant enzyme family of SOD is considered to be the first line of defense against oxygen toxicity. SOD accelerates the reaction in which superoxides are converted to H_2_O_2_.^32^ In the present study, significant increase in erythrocytes SOD activity was observed after intoxication compared to baseline values. This finding suggests that increased erythrocytes SOD levels resulted in production and accumulation of intracellular H_2_O_2_ which results in increase of MDA. Lipid peroxidation has also been used as a marker of oxidative stress and in the present study, monensin administration produced significant but not consistent increase serum MDA levels. 

Glutathione related enzymes such as GPx act either directly or indirectly as antioxidants. In the present study there was significant decrease in the activity of GPx in serum of monensin exposed goats. The depression of activity of this enzyme activity reflects perturbation in normal oxidative mechanisms during monensin toxicity.

The findings of the present study suggested that monensin intoxication in goats led to cardiac, skeletal and renal damage and a wide range of biochemical abnormalities. Oxidative stress could be involved in its pathogenesis.
